# The complete chloroplast genome of *Vaccinium fragile* (Vacciniaceae), a shrub endemic to China

**DOI:** 10.1080/23802359.2019.1627948

**Published:** 2019-07-11

**Authors:** Wei Guo, Lei Luo, Yanyan Huang, Guohua Li, Xinhua Wang, Tiantian Cheng, Zhongkui Sun, Feng Wang, Lin Zhang, Wei Li

**Affiliations:** a Taishan Academy of Forestry Sciences, Taian, Shandong, P. R. China;; b Taian Time Garden Technology Development Co., Ltd, Taian, Shandong, P. R. China;; c College of Landscape Architecture and Forestry, Qingdao Agricultural University, Qingdao, Shandong, P. R. China

**Keywords:** *Vaccinium fragile*, chloroplast genome, Illumina sequencing

## Abstract

*Vaccinium fragile*, an endemic species in China, is a plant of the family Vacciniaceae. It is an evergreen shrubby tree distributed in Sichuan, Guizhou, Yunnan, and Tibet province of China. The chloroplast (cp) genome of *V. fragile* is 169,480 bp in size containing 123 unique genes, including 8 rRNA genes, 38 tRNA genes, and 77 protein-coding genes (PCGs). Phylogenetic analysis exhibited that both *V. macrocarpon* and *V. fragile* were phylogenetically closer to *Arbutus unedo* than other taxa in this study.

*Vaccinium fragile* is a plant of the family Vacciniaceae. It is an evergreen shrubby tree and reaches a height of 1.5 m. It is distributed in Sichuan, Guizhou, Yunnan, and Tibet province of China (Fang and Huang 1997). It is an acid soil indicator and grows in pine forest and hillside shrubbery. The altitude of its natural mountain habitat is between 1100 and 3400 m. The taste of the ripe edible berry ranges from sour to sweet. In traditional Chinese medicine, the roots and leaves of *V. fragile* showed antipyretic and antidotal effects. The genomic sequence information is urgently needed to promote molecular evolution, systematics research, conservation, and utilization of *V. fragile*. The objectives of the present study were to reconstruct the cp genome of *V. fragile* to provide genetic information resource.

Leaves were sampled from a mature *V. fragile* tree at Jiuhe, Yulong Naxi Autonomous County, Lijiang, Yunnan, China (26.37°N, 99.56°E) and chilled with liquid nitrogen immediately. The voucher specimen (accession no. JH_2019_Yulong_Taian) was stored at –80 °C in Taishan Academy of Forestry Sciences (TSAF). Genomic DNA (gDNA) was obtained from homogenized leaf tissues using a modified CTAB protocol (Doyle and Doyle 1987). The quantity and quality of the purified gDNA were detected by Nanodrop 8000 and via the Agilent 2100 Bioanalyzer. A library with 350 bp fragments inserted was constructed with 1 μg purified DNA and high-throughput sequenced with paired end (PE) reads of 2 × 150 bp on Illumina Hiseq 2500 platform. Raw reads were filtered and trimmed to remove low quality and contaminated reads by trim_galore v0.4.4. Total 8.5 Gb of clean data were aligned to the *V. macrocarpon* complete cp genome (GenBank no. JQ757046) as a reference using bowtie2 v2.2.4 (Langmead and Salzberg 2012) and assembled with SPAdes v3.10.1 (Bankevich et al. 2012). The final cp genome was annotated using ARAGORN v1.2.38 (Laslett and Canback 2004), DOGMA (Boore et al. 2004), and HMMER 3.1b2 (Finn et al. 2011).

The cp genome of *V. fragile* (GenBank no. MK816301) is 169,480 bp in size with total AT content 63.2%. It contains a 2963 bp small and 105,767 bp large single-copy regions with AT contents 70.9 and 64.1%, respectively, and two 30,375 bp inverted repeat regions with AT content 61.1%. In the cp genome of *V. fragile,* there are 123 unique genes, including 8 rRNA genes, 38 tRNA genes, and 77 PCGs. Eight genes harbour one intron each, while no gene harbours two introns.

To perform the molecular phylogenetic analysis, 15 published complete cp genomes were aligned by MAFFT v7.307 (Katoh and Standley 2013). Finally, a maximum likelihood (ML) tree was constructed using RAxML v.7.2.6 with 1000 bootstraps under the GTRGAMMA model (Stamatakis 2006). The ML phylogenetic tree showed that both *V. macrocarpon* and *V. fragile* were phylogenetically closer to *Arbutus unedo* than other taxa in this study (Figure 1), which was consistent with the most recent report (Bao 2018). Most nodes in the cp genome ML tree were strongly supported.

**Figure 1.**
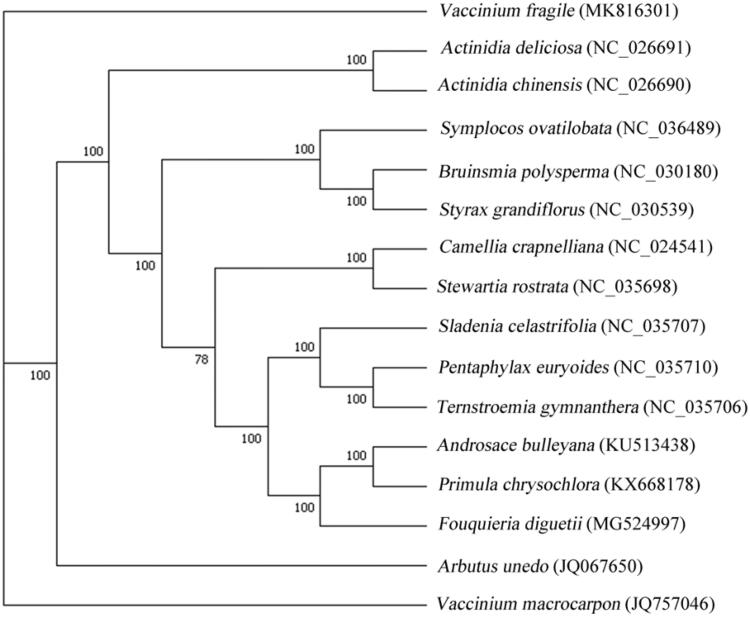
Phylogenetic tree based on 16 complete cp genome sequences. The bootstrap support values are shown next to the branches.

## References

[CIT0001] Bankevich A, Nurk S, Antipov D, Gurevich AA, Dvorkin M, Kulikov AS, Lesin VM, Nikolenko SI, Pham S, Prjibelski AD, et al. 2012 SPAdes: a new genome assembly algorithm and its applications to single-cell sequencing. J Comput Biol. 19:455–477. 22506599 10.1089/cmb.2012.0021 PMC3342519

[CIT0002] Bao WH 2018 A phylogenetic study of Actinidiaceae based on chloroplast genomes [master’s thesis]. Wuhan: University of Chinese Academy of Sciences.

[CIT0003] Boore JL, Jansen RK, Wyman SK 2004 Automatic annotation of organellar genomes with DOGMA. Bioinformatics. 20:3252–3255. 15180927 10.1093/bioinformatics/bth352

[CIT0004] Doyle JJ, Doyle JD 1987 A rapid DNA isolation procedure for small quantities of fresh leaf tissue. Phytochem Bull. 19:11–15.

[CIT0005] Fang RZ, Huang SH, 1997 Vacciniaceae In: Ma EW, Zhou SQ, Zhang XS, Wang WC, Chen JR, Zhang HD, Lin WT, Yang CY, Lai SK, Huang SQ, et al. editors. Sylva sinica. Beijing (China): China Forestry Publishing House; p. 3324–3341.

[CIT0006] Finn RD, Clements J, Eddy SR 2011 HMMER web server: interactive sequence similarity searching. Nucleic Acids Res. 39:W29–W37. 21593126 10.1093/nar/gkr367 PMC3125773

[CIT0007] Katoh K, Standley DM 2013 MAFFT multiple sequence alignment software version 7: improvements in performance and usability. Mol Biol Evol. 30:772–780. 23329690 10.1093/molbev/mst010 PMC3603318

[CIT0008] Langmead B, Salzberg SL 2012 Fast gapped-read alignment with Bowtie 2. Nature Methods. 9:357. 22388286 10.1038/nmeth.1923 PMC3322381

[CIT0009] Laslett D, Canback B 2004 ARAGORN, a program to detect tRNA genes and tmRNA genes in nucleotide sequences. Nucleic Acids Res. 32:11–16. 14704338 10.1093/nar/gkh152 PMC373265

[CIT0010] Stamatakis A 2006 RAxML-VI-HPC: maximum likelihood-based phylogenetic analyses with thousands of taxa and mixed models. Bioinformatics. 22:2688–2690. 16928733 10.1093/bioinformatics/btl446

